# The formulation and characterization of 3D printed grafts as vascular access for potential use in hemodialysis†

**DOI:** 10.1039/c8ra01583j

**Published:** 2018-04-24

**Authors:** Bill Cheng, Yue-Min Xing, Nai-Chia Shih, Jen-Po Weng, Hsin-Chieh Lin

**Affiliations:** Department of Materials Science and Engineering, National Chiao Tung University Hsinchu 30010 Taiwan Republic of China hclin45@nctu.edu.tw

## Abstract

Arteriovenous graft (AVG) failure continues to be a life-threatening problem in haemodialysis. Graft failure can occur if the implanted graft is not well-matched to the vasculature of the patient. Likewise, stenosis often develops at the vein-graft anastomosis, contributing to thrombosis and early graft failure. To address this clinical need, a novel ink formulation comprised of ACMO/TMPTA/TMETA for 3D printing a AVG was developed (ACMO-AVG), in which the printed AVG was biocompatible and did not induce cytotoxicity. The ease of customizing the ACMO-AVG according to different requirements was demonstrated. Furthermore, the AVG displayed similar mechanical properties to the commercially available arteriovenous ePTFE graft (ePTFE-AVG). Unlike ePTFE-AVG, the ACMO-AVG displayed excellent anti-fouling characteristics because no plasma protein adsorption and platelet adhesion were detected on the luminal surfaces after 2 h of incubation. Similarly, exposure to human endothelial cells and human vascular smooth muscle cells did not result in any cell detection on the surfaces of the ACMO-AVG. Thus, the present study demonstrates a newly developed 3D printing ink formulation that can be successfully 3D printed into a clinically applicable vascular access used for haemodialysis.

## Abstract 2

An arteriovenous graft that was successfully 3D printed with a novel printing ink formulation that displayed excellent mechanical and anti-fouling properties.

## Graphical abstract

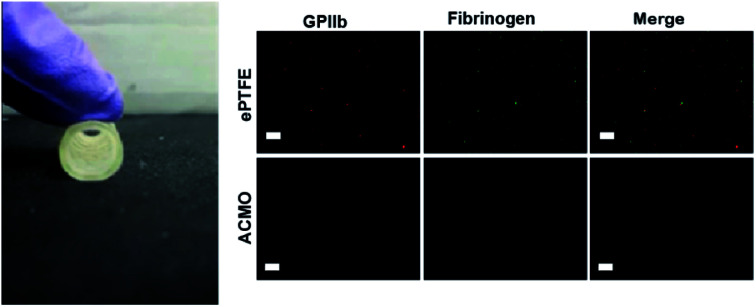

## Introduction

Globally, the number of patients with end-stage renal disease (ESRD) continues to rise.^1^ Apart from kidney transplant, dialysis is the only other treatment available to these patients. There are two different dialysis methods, peritoneal dialysis and haemodialysis, with the latter most commonly applied to ESRD patients. The majority of ESRD patients receive haemodialysis two to three times a week.^2^ Patients that require haemodialysis are implanted with either an arteriovenous fistula (AVF) or an arteriovenous graft (AVG) under the skin that bridges an artery and a vein, providing needle placement access for haemodialysis.^3^ Clinically, the AVF is the preferred method when compared to AVG since the former method has a relatively low risk of long-term complications and better long-term patency. However, AVF placements are susceptible to early failure and have a 20–60% chance of not providing enough flow enhancement to facilitate sufficient dialysis. It has been estimated that the early failure rate of AVF placements is 33.8%.^4^

Maturation failure is the most common reason that contributes to the early failure of AVFs, in which patients will need to switch to AVGs until a suitable AVF is available. Unlike AVFs, AVGs enable quicker vascular access in haemodialysis patients since cannulation can start within two to four weeks after placement as opposed to months with AVF placements. Synthetic AVGs such as expanded polytetrafluoroethylene (ePTFE) have been clinically used for decades, but can only be applied to vessels with large diameters (≥6 mm).^5^ This is a drawback for AVG placements since a significant number of ESRD patients only have smaller vessels available for graft implantation. Grafts with unsuitable diameters can cause vascular steal in the adjacent vessels, which can lead to stenosis.^6^ Furthermore, immediate stenosis may happen if twisting and kinking occurs during the graft's placement. If the graft needs to be placed in a looped configuration, tunnelling the loop too tight can lead to kinking at the apex of the graft.^7^ Therefore, it is ideal if the design and fabrication of the graft can be customized according to the vasculature of each individual patient, preventing the occurrence of twisting and kinking.

In addition to improper placement, the development of intimal hyperplasia (IH) is another major cause of AVG failure. Studies have shown the graft function of ePTFE vascular grafts in ESRD patients at 12 months after surgery varies from 10% to 78% because of IH.^8^ IH not only results in haemodialysis failure, it can potentially cause mortality. Although attempts have been made to modify the surfaces of ePTFE through the addition of heparin^9^ or nitinol,^10^ the clinical results indicate that these modifications can only prevent the development of IH at an early stage after the initial graft placement. It has been estimated that most haemodialysis patients require graft replacement 12 months after the initial graft implantation.^11^ Since a significant number of haemodialysis patients are >65 years old, constant graft replacement creates a significant toll on their health and increases the chance of bacterial infection. Thus, in addition to a fabrication method that allows physicians to custom-design AVGs with minimum technical skill, there is an urgent need to develop a novel material with properties that minimize the chance of developing IH.

A recent study has demonstrated electrospinning as an effective method to prepare custom-designed AVGs.^12^ Similarly, human acellular vessels have also been demonstrated to be viable option to replace the commercial arteriovenous ePTFE graft (ePTFE-AVG).^13^ However, either method will require an experienced technician and expensive equipment. It is believed 3D printing is the future for fabricating custom-designed AVGs that will meet all the needs of a haemodialysis patient. In the present study, the physical and biological characteristics of a novel designed AVG have been investigated, in which the graft was printed with a novel printing ink formulation using a 3D printer. The physical and mechanical properties of the AVG were analysed, in which the tensile strength and suture retention strength were measured. In addition, the 3D printed graft was examined for its anti-fouling effects against plasma proteins, human platelets and human endothelial and vascular smooth muscle cells.

## Results and discussion

Depending on the configuration of the 3D printer available, there are limitations to what materials can be 3D printed. In the present study, the chosen 3D printer utilizes digital light processing (DLP) technology, in which the printed materials are solidified when exposed to a near UV light source. In particular, acrylic polymers are the materials commonly used as the printing materials for DLP due to their rapid curing reactions and high resolution optical sources, in which the materials become solidified through the establishment of acrylic bonds after being exposed to UV-light.^14^ Already, there have been numerous studies demonstrating the potential of 3D printed acrylic polymers for different biomedical applications.^15^ Since the 3D printed AVG needs to have viscoelastic and anti-fouling properties comparable to the ePTFE-AVG, the different acrylic polymers that can be 3D printed were evaluated. It was decided that two different 3D printing ink formulations would be examined for their applications as vascular access, 4-acryloylmorpholine (ACMO) and PEGDA (PEGDA575/250) (Fig. S1†). Polymerized ACMO is a hydrophilic polymer and is known to be non-toxic, non-antigenic and biocompatible.^16,17^ Moreover, it shows a repellent property for protein adsorption and has been demonstrated as an excellent material for making anti-fouling membranes.^18^ TMPTA and TMETA are cross-linkers that will facilitate rapid acrylic bond formation, thus providing the necessary mechanical strength and viscoelasticity.^19^ Likewise, PEGDA is an acrylic polymer that is known for its excellent anti-fouling properties and biocompatibility.^20^ However it is also commonly known to be a material that is very hard and brittle after being 3D printed.^21^

### The design and fabrication of ACMO-AVG

**Fig. 1:**
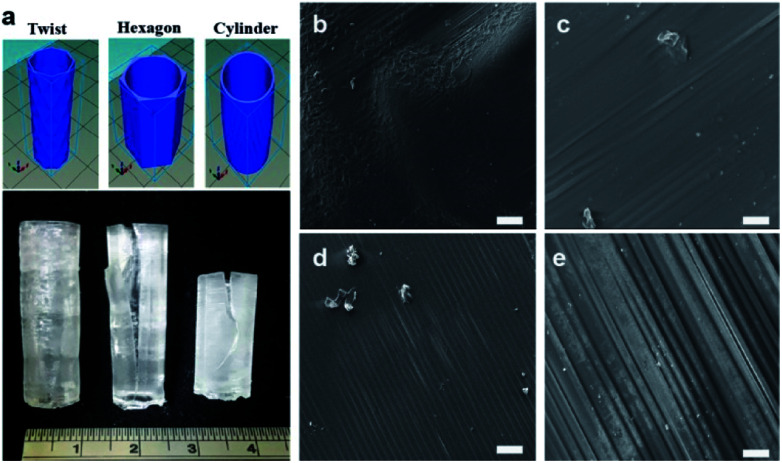
(a) The CAD design of the ACMO-AVG with different outer rim designs and the corresponding 3D printed grafts; scale bar, cm. The SEM images of the ACMO-AVG with the (b and c) twisted design or the (d and e) cylindrical design for the outer rim. Scale bar, 100 μm for (b) and (d) and 10 μm for (c) and (e).

Using computer-aided design (CAD) software, different designs of the arteriovenous ACMO graft (ACMO-AVG) were proposed (Fig. 1). Just like any other 3D printed object, the ACMO-AVG will need to withstand a certain amount of cross-section compression stress while being 3D printed.^22^ It was noticed that the structural integrity of the printed AVG was greatly influenced by the design of the outer rim (Fig. 1a). Unlike the ACMO-AVG with the twisted design, splitting occurred in the AVGs that were 3D printed with either a hexagonal or cylindrical design (Table S1†). Since conventional software was used, the CAD models that were drawn could not be converted into perfect cylinders.^23^ It was, instead, approximated into a cluster of triangles, where the uneven lines formed sites that were easy for crack propagation, which can lower the mechanical strength of the printed ACMO-AVG. To overcome this challenge, a hexagonal outer rim with a smooth surface was designed. However, since the edges also formed propagation sites for fractures, the hexagonal structure was spun to form a twisted-like structure. After adjusting the spacing between each spiral, it was decided that the hexagonal spiral with a sixty-degree increment gave the best mechanical strength. Likewise, the arteriovenous PEGDA graft (PEGDA-AVG) was structurally stable after being 3D printed with twisted outer rim (Fig. S2a†).

To further understand how the twisted outer rim maintained the structural integrity of the ACMO-AVG, the surface structure of the graft (Fig. 1b and c) was examined and compared to the cylindrical ACMO-AVG (Fig. 1d and e). Evidently, the twisted ACMO-AVG displayed a smoother surface with less signs of layer splitting when compared to the cylindrical ACMO-AVG. In contrast, the layer splitting was still noticed on the surface of the PEGDA-AVG with twisted outer rim (Fig. S2b–d†). Layer splitting is a common problem in 3D printing and bioprinting,^24^ which can occur when one layer fails to bond correctly with the upper or lower layer. Apart from an impropriate design, factors such as the polymer composition, room temperature and printing speed can also contribute to layer splitting.^25^ Since it is desirable to have an AVG ready within the shortest possible timeframe, the speed setting of the 3D printing protocol allowed a 20 cm ACMO-AVG to be prepared in 12 h. It is possible to 3D print the ACMO-AVG with a cylindrical outer rim without layer splitting, although the fabrication time would be significantly increased. Nevertheless, it was clear that the ACMO-AVG with the twisted outer rim design was structurally more stable than the other designs and that it had less signs of layer splitting when compared to PEGDA-AVG. Consequently, the ACMO-AVG was the main focus for the subsequent experiments.

Small vein diameter has been one of main factors that reduce the patency of AVFs. It is also the reason that limits some AVG placements because an unmatched graft diameter may create turbulent blood flow, creating thrombotic events.^26^ The customizability of the 3D printed ACMO-AVG was demonstrated by showing the diameter of the graft could be easily adjusted to either 4 or 6 mm (Fig. S3†). Since it is difficult for the current manufacturing method to customize the design of AVGs according to the vasculature of an individual patient,^27^ the future application of 3D printing in AVG fabrication is inevitable. Moreover, 3D printing will enable the rapid adjustment of other key physical features, such as the length and wall thickness of AVGs.

### Mechanical analysis of the ACMO-AVG

**Fig. 2:**
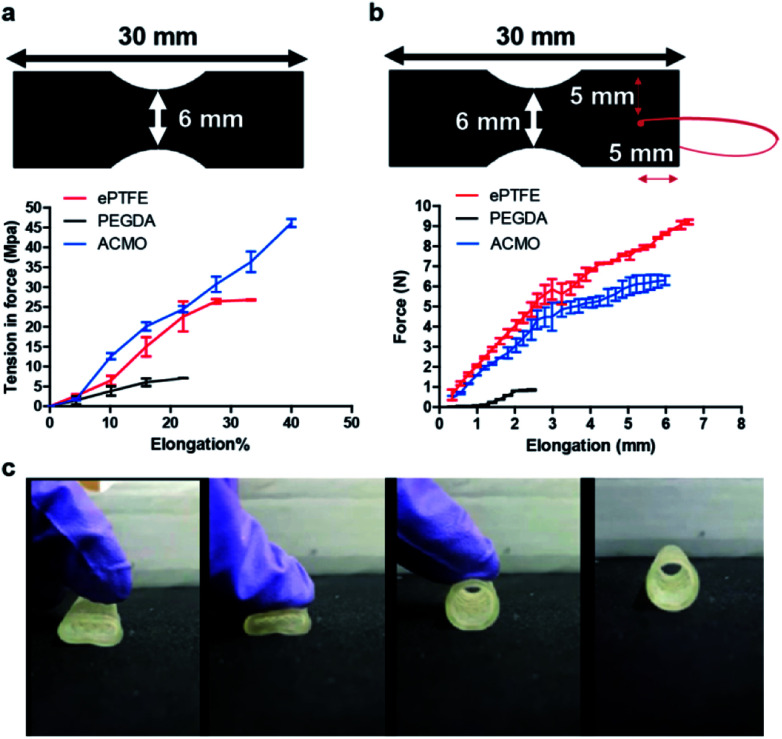
(a) The 3D printing ink formulation used for fabricating either the ACMO-AVG or PEGDA-AVG was 3D printed into the shape represented by the schematic used in the dynamic mechanical analysis (*n* = 3). (b) The suture retention strength analysis for each tested sample was determined by measuring the required force to pull the 5-0 suture through the sample (*n* = 3). (c) The elasticity of the ACMO-AVG was demonstrated by thumb pressing.

Clinically, the elasticity of an AVG is critical as the graft will be stretched to bridge a vein and an artery. Therefore, the tensile strength of the ACMO-AVG was examined using a dynamic mechanical analyser, in which the printing ink formulation of the ACMO-AVG was 3D printed into the sample shape as specified by the American Society of Testing and Materials for mechanical testing of polymers (ASTM-D4092).^28^ The printed sample was loaded horizontally into textured grips and was pulled from both ends simultaneously at the rate of 0.1 mm s^−1^ until the graft snapped. The result was compared to a trimmed strip of the ePTFE-AVG and the 3D printed PEGDA sample (Fig. 2a). Consequently, the 3D printed PEGDA sample snapped very quickly under a very low tension force. Notably, the 3D printed ACMO sample displayed a better tensile strength and higher maximum elongation when compared to ePTFE, suggesting the 3D printed ACMO-AVGs would have an elastic property that is comparable to ePTFE-AVG. ACMO has been previously demonstrated to enhance the elasticity when included in copolymer composites.^29^ Also, due to its superior elasticity, ACMO has been applied in the fabrication of touch screen panels.^30^

To ensure the ACMO-AVG can withstand the surgical manipulation experienced during implantation, the suture retention strength of the samples printed with the ink formulation of the graft was analysed. Out of the three analysed graft materials, it was clear that the ePTFE sample had the best suture retention strength when compared to the other two 3D printed samples (Fig. 2b). Unlike the ePTFE sample, it was noticed the penetration of the suture created a significant crack in the PEDGA sample and a smaller crack in the ACMO sample. This indicated that although the ACMO sample had better tensile strength measurement than ePTFE, the 3D printed sample was, however, still too brittle for needle penetration. The strong suture retention strength displayed by ePTFE was likely due to the yarn that was wrapped externally around the ePTFE-AVG, which has been demonstrated to enhance the suture retention strength of the commercial graft.^31^ Similar results were also obtained by calculating the fracture energy of the three AVGs (Table S2†). Thus, the printing ink formulation of the ACMO-AVG will need to be further modified in order to improve its suture retention strength for future clinical applications.

Since the average pressure in arterial circulation is 100 mm Hg and the hydrostatic pressure in an AVG can reach up to 250 mm Hg, it was decided to see whether the ACMO-AVG could be restored to its original shape after pressure was applied to the graft. If the graft failed to return to its original shape after pressure was applied, then the graft would likely be ruptured in a clinical setting. As expected, the ACMO-AVG recovered its original shape after the pressure was lifted (Fig. 2c). It is understood that the method used to measure the pressure strength of the 3D printed graft was quite preliminary, a more sophisticated pressure recording method is warranted for future study.^32^ Nevertheless, in conjunction with the data obtained from the tensile testing, it is clear that the ACMO-AVG has excellent mechanical properties because the ACMO sample displayed better tensile strength than the ePTFE sample and recovered its original shape after the pressure was lifted.

### Structural integrity and toxicity analysis

**Fig. 3:**
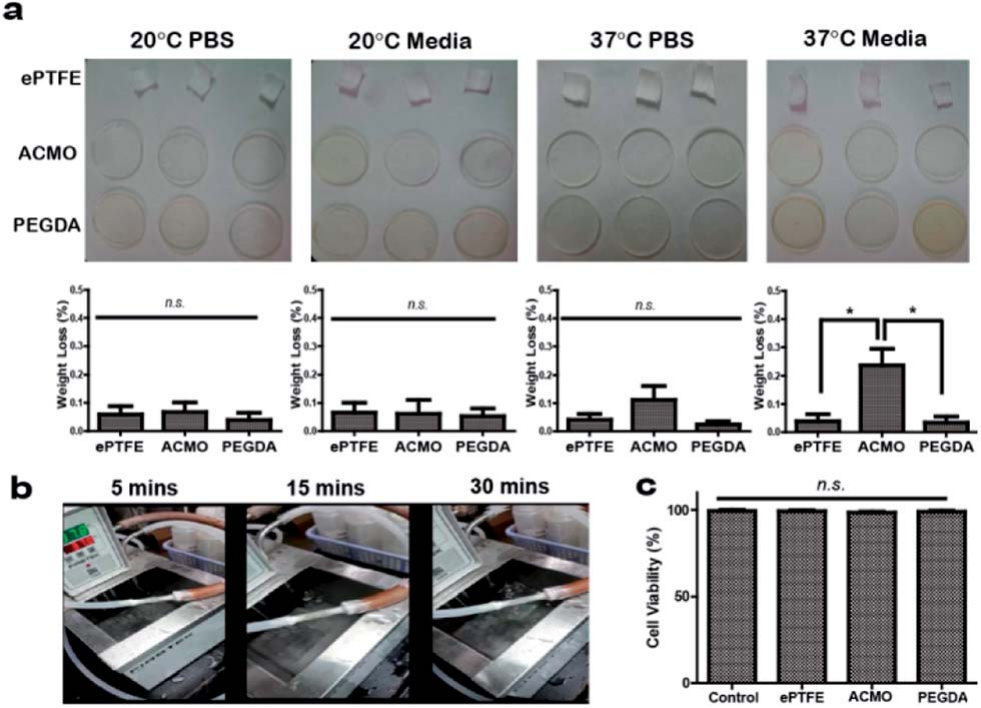
(a) The ink formulation of either ACMO-AVG or PEGDA-AVG 3D printed into a disc; the weight of the disc was measured before and after being incubated at either 20 °C or 37 °C in either PBS or cell culture media for 48 h. The amount of weight loss is presented as weight loss (%) and compared to a strip of commercial ePTFE-AVG; n.s., not significant and *P < 0.05. (b) The ACMO-AVG placed in between the conduits of a heated circulating water bath, in which water (∼37 °C) was circulated through the 3D printed graft for 30 min. (c) The percentage of viable WS1 cells after being incubated in the culture media pre-exposed to the different graft materials for 72 h. The cell viability was determined using a haemocytometer and the results were compared to the WS1 cultured in normal media (control); n.s., not significant.

3D printed products that are applied in biological fluids such as blood often face the challenge of losing their original structure because of swelling.^33^ Although in cases such as drug delivery, the breakdown of a swollen 3D printed implant can lead to a beneficial effect, *e.g.* controlled drug released.^34^ However, for 3D printed AVGs it would be more desirable if the graft maintains its original structure after a long period of exposure to biological fluid. Moreover, the breakdown of AVGs can also accelerate the development of IH, as noticed in many synthetic AVGs.^35^ To demonstrate the 3D printed ACMO-AVG was structurally stable at room temperature and physiological temperature, the graft formulation was 3D printed into a disc shape and submerged in either PBS or FBS containing culture media at either ∼20 °C or 37 °C for 48 h (Fig. 3a). The weight of the disc was measured before and after the incubation period at either temperature, and the results were compared to a trimmed strip of ePTFE-AVG and a 3D printed PEGDA disc. The differences in the percentage weight loss at ∼20 °C between the three samples were insignificant. A significant difference was noticed when the printed ACMO disc was incubated in the 37 °C culture media when compared to the other two tested samples. However, the percentage weight loss was minimal. Thus, the data indicated that the acrylic bonds established within the ACMO/TMPTA/TMETA mixture were stable enough to avoid any significant degradation of the 3D printed material.

The structural stability of the ACMO-AVG was further evaluated by connecting the graft between the two conduits of a heated circulating water bath (Fig. 3b). The temperature of the water bath was set at 37 °C and the flow rate was set at 10 L min^−1^, in which the water was allowed to flow through the graft for at least 30 min. During 30 min of circulation, the graft remained intact and did not display any leakage. Since the required blood flow for a reliable vascular access has been estimated to be 1.3 to 1.9 L min^−1^,^36^ the result of the water flow test indicated that the ACMO-AVG was able to sustain water pressure from flow rate that was ∼5 to ∼7 times higher than the physiological blood flow rate. Undoubtedly, given the viscosity of blood is to different to water and that the presence of blood components can also influence the structural integrity of the ACMO-AVG, animal studies are warranted to further investigate the durability of the ACMO-AVG when exposed to dynamic blood flow.

Toxicity has been one of the key factors that have limited the clinical application of 3D printed devices. Although ACMO has been approved by the US Food and Drug Administration (FDA) for some medical applications^37–39^ and that TMPTA and TMETA have been studied extensively for different 3D printed biomedical products,^40^ a photoinitiator, on the other hand could induce cell toxicity. The main purpose of a photoinitiator is to create free radicals when exposed to UV light, which facilitate acrylic bond formation during the 3D printing process. Although the 3D printing protocol in the present study included an post-UV curing step to remove the trace amounts of the photoinitiator, cytotoxicity tests are needed since trace amounts of the photoinitiator could cause damage to the cell membrane and disrupt nucleic acid and protein synthesis.^41^ To evaluate the toxicity of the ACMO-AVG, a media extraction method was conducted according to ISO 10933.^42^ The 3D printing ink formulation used for the ACMO-AVG was printed into a disc, which was submerged in the cell culture media used for human skin fibroblasts (WS1) for 72 h at 37 °C. Subsequently, the pre-exposed media was removed from the 3D printed material and used to culture WS1 for another 72 h at 37 °C. When compared to the positive control, which was WS1 cultured in normal media, the cell viability of the cells cultured the different pre-exposed media all showed similar cell viability (Fig. 3c and S4†). The data indicated that the established 3D printing protocol was effective in removing any traces of PI-819 and demonstrated the 3D printed ACMO-AVG had good biocompatibility. Even though photoinitiators are a cause for safety concerns for many 3D printed medical implants, efforts have already been made to prepare nano-photoinitiators with improved biosafety.^43^

### The anti-fouling characteristics of the ACMO-AVG

**Fig. 4:**
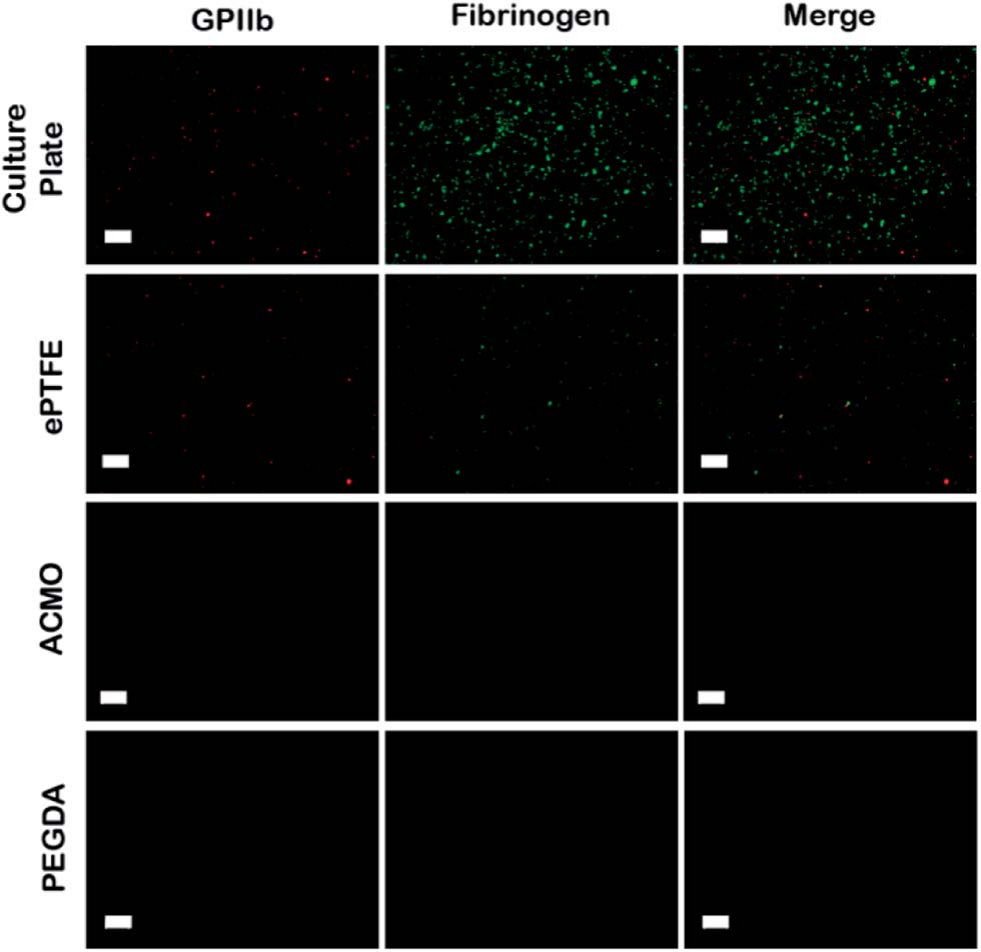
The luminal surfaces of the trimmed strips of ePTFE-AVG, ACMO-AVG and PEGDA-AVG exposed to outdated human PRP for 2 h at 37 °C with shaking. The adhered platelets and the adsorbed fibrinogen were probed with anti-human GPIIb (red) and anti-human fibrinogen antibodies (green), respectively. Scale bar, 50 μm.

Thrombosis is a major contributor in the development of IH that ultimately leads to graft failure. Clinically, thrombosis begins with plasma protein deposition onto the synthetic surfaces, which then mediate the subsequent platelet adhesion and activation events.^44^ Activated platelets release cytokines that recruit immune cells and amplify the coagulation cascade. Consequently, it has become a clinical standard that the luminal surfaces of AVGs need to prevent fouling by the plasma proteins.^44^ To evaluate the anti-fouling property of ACMO-AVG, the graft was cut normal to the long axis into small strips dimension of 20 mm × 5 mm. The luminal surfaces of the trimmed strips were then exposed to outdated human platelet rich plasma (PRP) at 37 °C for 2 h with shaking. The unbound platelets and plasma proteins were washed off with PBS and the anti-fouling characteristic assessed using anti-human GPIIb and anti-human fibrinogen antibodies (Fig. 4).

It has been well-demonstrated the adhesion of platelets on a surface is mediated by the proteins adsorbed on that surface.^45^ Therefore, as more plasma proteins are adsorbed on the analysed samples, the higher the number of adhered platelets was expected. Accordingly, strong signals of anti-human GPIIb and anti-human fibrinogen antibodies were detected in the human PRP exposed tissue culture plate (Fig. 4). A weaker signal of either anti-human GPIIb or anti-human fibrinogen antibodies was seen in the human PRP coated ePTFE AVG. In contrast, the signals for human GPIIb and fibrinogen were not detected in either the human PRP exposed ACMO-AVG or PEGDA-AVG. Evidently, the data indicated the luminal surface of the ACMO-AVG had similar anti-fouling effects as PEGDA-AVG and the ACMO-AVG can minimize platelet adhesion more effectively than the ePTFE AVG. Similar results have also been reported in other studies on platelet adhesion on commercial ePTFE AVGs, where platelet adhesion developed on the uncoated AVG but not on a heparin-coated ePTFE AVG.^46^ However, several clinical studies on heparin-coated or other material coated ePTFE all showed these coatings could only allow minimal platelet adhesion in the first 5 months after their initial placement.^47^ Although it is not understood why so many ePTFE-AVGs with modified luminal surfaces have unsatisfying long-term performance, it is becoming clear that the strategy in coating the luminal surfaces of ePTFE-AVG can only exert minimal anti-fouling effects.

### The inhibition of endothelial cells and smooth muscle cell adhesion

**Fig. 5:**
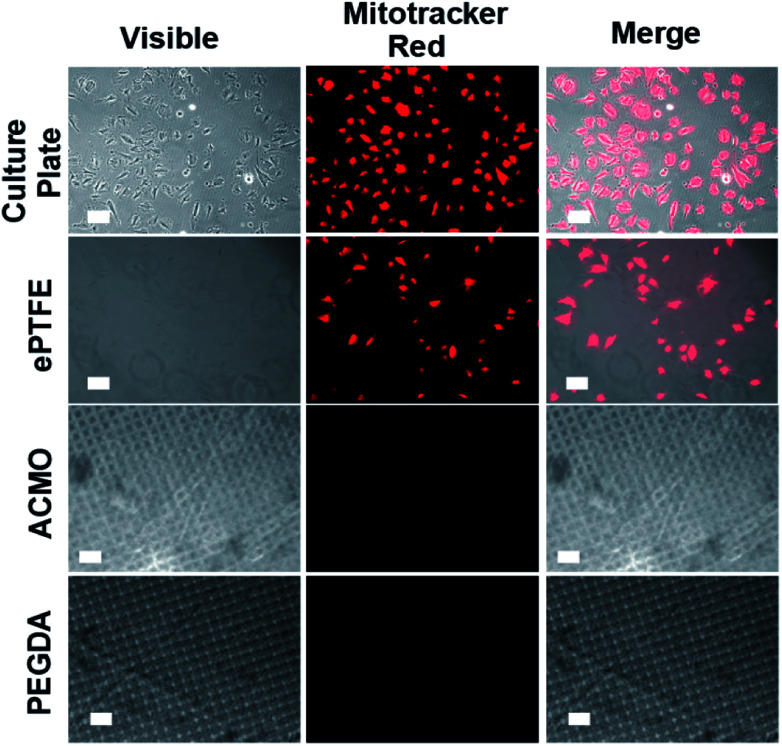
The exterior surface of the different AVGs exposed to HUVECs overnight at 37 °C. The adhered cells were stained with Mitotracker red (red) for 2 h at 37 °C, de-stained by PBS washing and then subjected to fluorescence imaging. Scale bar, 50 μm.

**Fig. 6:**
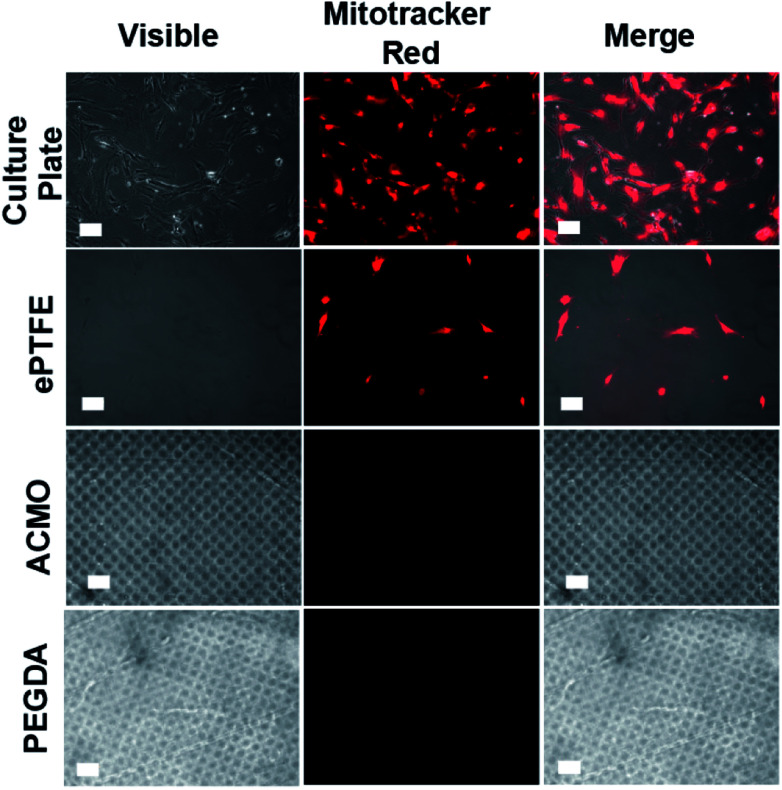
The exterior surface of the different AVGs exposed to hVSMCs overnight at 37 °C. The adhered cells were stained with Mitotracker red (red) for 2 h at 37 °C, de-stained by PBS washing and then subjected to fluorescence imaging. Scale bar, 50 μm.

In addition to thrombosis, endothelial cells (ECs) and vascular smooth muscle cells (VSMCs) also promote the development of IH in AVGs. The uncontrolled growth of ECs and VSMCs at the graft-vein anastomosis leads to IH development at the connected junction, which accelerates graft failure.^48^ To examine whether the ACMO-AVG could promote EC and VSMC adhesion, the exterior surfaces of trimmed strips of the graft were exposed to human umbilical vascular endothelial cells (HUVECs) and human vascular smooth muscle cells (hVSMCs) overnight at 37 °C. When compared to the number of HUVECs detected on the cell culture plate, a lower number of cells was detected on the surface of the ePTFE-AVG (Fig. 5). Both the ACMO-AVG and PEGDA-AVG did not show any sign of cell growth on their surfaces, suggesting their anti-fouling property could repel HUVECs from attaching to their surfaces. Moreover, the results indicated that the twisted design of the outer rim of either 3D printed AVG did not promote cell adhesion. Similar results were also noticed in the cell adhesion study with hVSMCs, in which minimal cell adhesion was detected on the exterior surface of the ePTFE-AVG when compared to the cell culture plate control and no cells were detected on the exterior surfaces of either ACMO-AVG or PEGDA-AVG (Fig. 6).

It has been envisaged that IH could be minimized if the implanted graft was rapidly covered with endothelial cells, thus preventing thrombosis and immune recognition.^49^ A study in a porcine model showed that ePTFE-AVG coated with anti-CD34 antibodies, which are antibodies that specifically target endothelial progenitor cells, allowed at least 85% of the graft surface to be covered with ECs within 3 days of implantation.^50^ However, when compared to the uncoated ePTFE-AVGs, which only had 32% of the graft surfaces covered with ECs, IH at the venous anastomosis was strongly increased in the anti-CD34–coated grafts. This indicated that rapid endothelisation accelerates the development of IH in implanted AVGs rather than preventing or minimizing the pathological process. Likewise, ePTFE-AVG coated with either anti-thrombotic peptides^51^ or heparin^52^ did not display any significant improvement in down-regulating IH when compared to the uncoated graft. Therefore, it is clinically advantageous that the core material components of the graft exhibit strong anti-fouling characteristics (*e.g.* ACMO) rather than depending on an additional coating for its anti-fouling effects.

Recently, there have been numerous reports on developing tissue engineered vascular grafts (TEVGs) for haemodialysis.^53,54^ Contrary to the synthetic AVGs, the re-cellularisation of TEVGs with ECs and VSMCs did not result in the extensive development of IH or an immunogenic response at 6 months after the implantation in large animal models. Moreover, since the TEVGs consist of secreted extracellular matrix (ECM) proteins, the mechanical properties are similar to those in a recent phase II clinical trial; TEVGs developed with the VSMCs derived from deceased organs and tissue donors had a 12 months primary patency of 73%, which was similar to the ePTFE controlled group and did not display any post-cannulation bleeding after dialysis.^55^ Even though TEVGs seem to be a viable alternatives to ePTFE-AVGs, the cost for making one TEVG is likely to be unbearable for most haemodialysis patients. Extensive genetic screening needs to be performed for each batch of donor cells, and a minimum of 8 weeks is required for pulsatile cyclic distension. Moreover, the customization of TEVGs according to an individual patient's vasculature is likely to be challenging, as opposed to the 3D printed ACMO-AVG reported in the present study.

## Conclusions

As the number of patients requiring haemodialysis continues to increase annually, the demand for custom-designed AVGs is becoming an important need. The present study demonstrates that a customized AVG can be easily fabricated using a standard desktop 3D printer using a novel 3D printing ink formulation comprised of ACMO/TMPTA/TMETA. The ACMO-AVG was demonstrated to have strong tensile strength and was thermo-stable under physiological conditions. Moreover, the ACMO-AVG was shown to be non-toxic to human cells and displayed anti-fouling characteristics that prevent plasma protein deposition, platelet adhesion and endothelialisation. It is envisioned that in the future every haemodialysis centre could utilize 3D printing technology to manufacture their own AVGs to meet the demands of their ESRD patients.

## Experimental

### 3D printing formulation

The 3D printing resin formulation used for the arteriovenous ACMO-based graft (ACMO-AVG) was comprised of 4 mL of 4-acryloylmorpholine (ACMO, Sigma Aldrich, CAS# 5117-12-4), 0.5 mL of trimethylolpropane triacrylate (TMPTA, Sigma Aldrich, CAS# 15625-89-5) and 0.5 mL of trimethylolpropane ethoxylate triacrylate (TMETA, Sigma Aldrich, CAS# 28961-43-5) in 1 mL of ethanol. Likewise, the 3D printing ink formulation of the arteriovenous PEGDA graft (PEGDA-AVG) was comprised of 60% PEGDA-575, 30% PEGDA-250 (Sigma Aldrich, CAS# 26570-48-9) and 10% ethanol. Bis(2,4,6-trimethylbenzoyl)-phenylphosphine oxide was used to as the photoinitiator in the printing process. Computer assisted design (CAD) models were first drawn and the different structural features were specified. The models were then sliced into multiple images using the manufacturers provided software. All the 3D printing processes were conducted using a DLP 3D printer. For the purpose of this study, the curing time for both the ACMO-AVG and PEGDA-AVG were kept the same and after the printing process, the grafts were first rinsed with ethanol before being subjected to a post-curing exposure to 365 nm UV light for 2 min to ensure the full extent of polymerization.

### Field emission scanning electron microscopy

The ACMO-based printing ink formulation was 3D printed in the dimensions of 5 mm × 5 mm × 5 mm. The samples were then cleaned with 75% ethanol and further exposed to UV light (405 nm, 6 W cm^−2^) for 5 min. Subsequently, all the samples were freeze-dried (FDM-2 model, UNISS) for 24 h to ensure all the water was removed. Prior to loading the samples into the field emission scanning electron microscope (JSM-6700F model, JOEL), the samples surfaces were coated with platinum gold (10 mA) for 60 s.

### Tensile strength measurements

Samples were 3D printed in the dimensions of 30 mm × 10 mm × 5 mm. Then, the samples were first wipe-cleaned with 70% ethanol before loading onto a dynamic mechanical analyser (Tytron™ 250 Microforce testing system). All the samples were loaded into textured grips horizontally and pulled from both directions simultaneously at the rate of 0.1 mm s^−1^ until failure.

### Suture retention strength measurements

One end of the 3D printed samples (30 mm × 10 mm × 5 mm) was connected to the textured grip of a dynamic mechanical analyser (Tytron™ 250 Microforce testing system), while the other end had a 5-0 prolene suture passed through and knotted 5 mm from its edge. The suture was pulled at a rate of 50 mm min^−1^, in which the force required to pull the suture through the samples was recorded as the suture retention strength.

### Fracture energy analysis

Fracture energy analyses were determined by integrating the area under the stress–strain curve. The end points for the integration are the points of fracture, which represent the overall toughness of the analysed samples and the energy absorbed by the materials before break. The unit was expressed as mJ mm^−3^ (milli-joules per milli-meter squared).

### 37 °C water flow test

The 3D printed ACMO-AVG (length = 20 cm, diameter = 6 mm) was connected to the outflow tubing of a circulating water bath and that the other end of the graft was connected to another tubing. Both connections were sealed with parafilm to ensure no water leakage occurred while the water was flowed through the graft. The circulating water bath was set at 37 °C with a flow rate of ∼9.8 L min^−1^.

### Human platelet rich plasma and mammalian cell culture

Batches of outdated human platelet rich plasma (PRP) were purchased from Taiwan blood service foundation (Hsinchu blood centre, Taiwan), in which any experimental works involved the use of human PRP were approved by the research ethics committee for human subject protection, National Chiao Tung University. Human umbilical endothelial cells (HUVECs, ATCC CRL-1730) were cultured in endothelial cell medium (ScienCell, Cat# 1001) and maintained at 37 °C under a 5% CO_2_ atmosphere. Human vascular smooth muscle cells (hVSMCs, ATCC CRL-1999) were cultured in M199 (Sigma Aldrich, Cat# M4530) with 10% foetal bovine serum (FBS) and maintained at 37 °C under a 5% CO_2_ atmosphere. The conditioned media used for both mammalian cell lines were changed every two days and passaged when the cell confluency reached ∼80%.

### Material degradation test and cell toxicity

Either the ACMO-based or PEDGA-based printing ink formulation was 3D printed into a disc shape (*r* = 0.8 cm), which could fit into a 24-well plate. Likewise, sections of ePTFE were excised from a commercial ePTFE-AVG into a size that fitted in the well of a 24-well plate. The 24-well plate containing the 3D printed ACMO sample, PEGDA sample and trimmed sections of ePTFE were filled with either DMEM containing 10% FBS or PBS and incubated at 37 °C under a 5% CO_2_ atmosphere for 72 h. Subsequently, the weight of the three materials were determined and compared to their original weights. To determine if the materials had any toxic effect on the cell viability, the 3D printed ACMO sample, PEGDA sample and trimmed sections of ePTFE were firstly incubated in DMEM containing 10% FBS at 37 °C under a 5% CO_2_ atmosphere. The media were then extracted and used to culture human skin fibroblast (WS1, ATCC CRL-1502) at 37 °C under a 5% CO_2_ atmosphere for 72 h. The cell viability was then determined using a hemocytometer to count the number of dead cells *versus* the number of live cells.

### Immunostaining

Outdated human PRP containing 1 × 10^6^ platelets per mL were seeded onto PEGDA575/250 (3 : 2), PEGDA575/250 (2 : 3) and a section of ePTFE in a 24-well plate for 2 h with agitation at 37 °C. The plate was then washed with Tyrode's buffer (1.8 mM CaCl_2_, 1 mM, MgCl_2_, 2.7 mM KCl, 136.9 mM NaCl, 0.4 mM NaH_2_PO_4_, 11.9 mM NaHCO_3_ and 5.6 mM glucose, pH 6.8) and the bound PRP fixed with 4% paraformaldehyde in PBS overnight at 4 °C. The presence of human fibrinogen was probed with anti-human fibrinogen antibodies (GeneTex, GTX26666), whereas human CD41 was probed with anti-human CD41 antibodies (GeneTex, GTX113758). Both antibodies were incubated with their respective targets overnight at 4 °C with agitation before the plate was washed with 0.1% PBST and probed with the respective secondary antibodies. The adhered HUVECs and hVSMCs were first washed with PBS twice and then stained with Mitotracker red (1 : 10 000, ThermoFisher, Cat# M7512) for 2 h at 37 °C. The samples were then washed with PBS before being subjected to fluorescence imaging.

## Conflicts of interest

There are no conflicts to declare.

## Supplementary Material

RA-008-C8RA01583J-s001
