# Supplementary material for: The formulation and characterization of 3D printed grafts as vascular access for potential use in hemodialysis
Source: RSC Adv. 2018 Apr 24;8(28):15471–9. doi: 10.1039/c8ra01583j (PMC9080031; doi:10.1039/c8ra01583j)
Supplement: RA-008-C8RA01583J-s001 [file RA-008-C8RA01583J-s001.pdf]

Electronic Supplementary Material (ESI) for RSC Advances.
This journal is © The Royal Society of Chemistry 2018

Supporting Information

Title: Formulation and characterization of 3D printed grafts as vascular access for potential use in hemodialysis

Bill Cheng, Yue-Min Xing, Nai-Chia Shih, Jen-Po Weng, Hsin-Chieh Lin*

Dr. Bill Cheng, Yue-Min Hsing, Nai-Chia Shih, Jen-Po Weng, Prof. H.-C. Lin*
Department of Materials Science and Engineering, National Chiao Tung University, Hsinchu, 30010, Taiwan, Republic of China
E-mail: hclin45@nctu.edu.tw

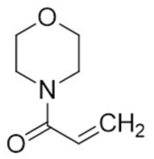

ACMO

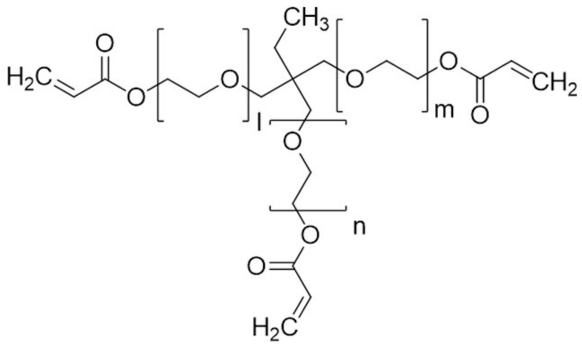

TMETA

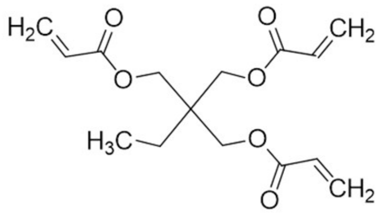

TMPTA

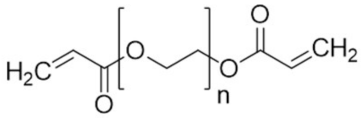

PEGDA

Figure S1. Chemical structures of the polymers used in fabricating the 3D printed AVG.

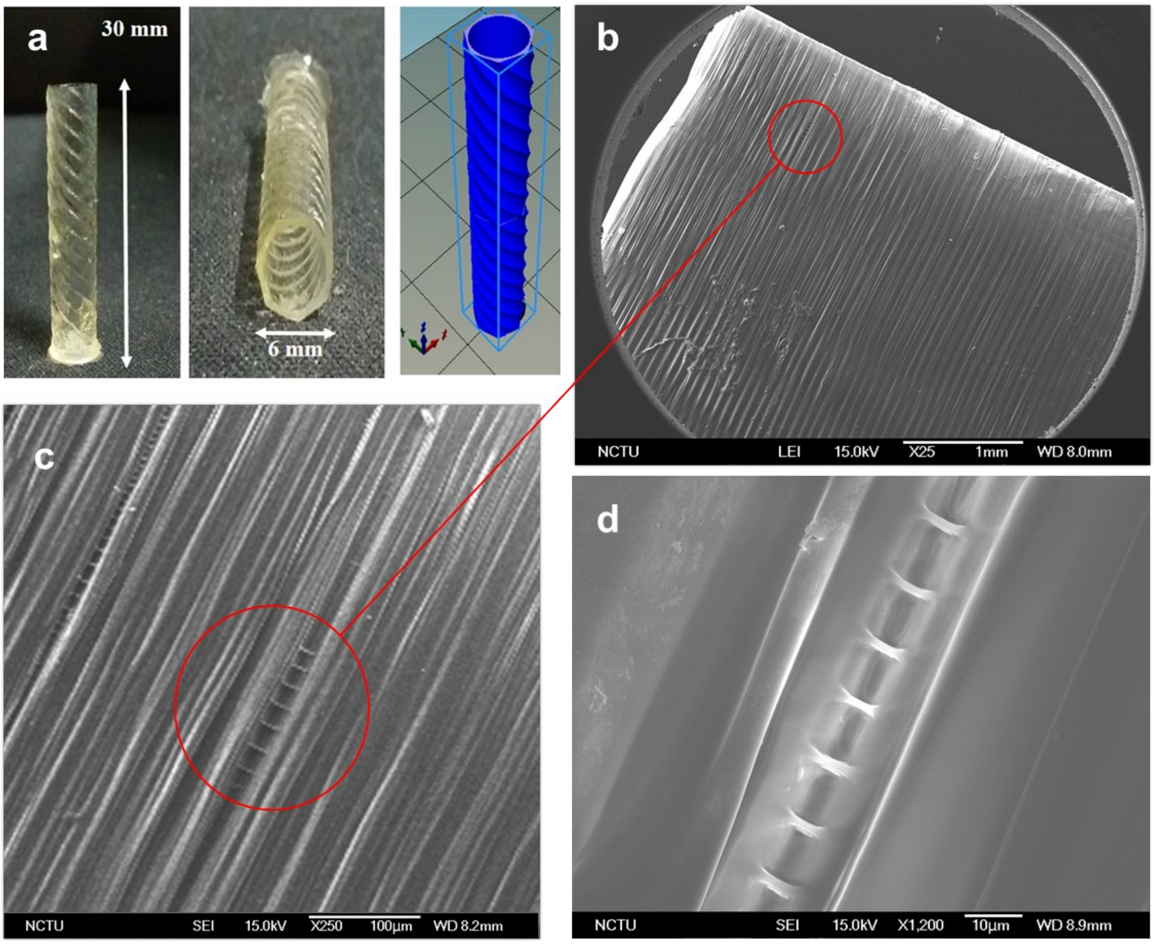

Figure S2. The PEDA ink formulation was 3D printed into (a) PEGDA-AVG with twisted outer rim. (b) SEM image at 25×; scale bar: 1 mm. (c) SEM image showed the small area contained layer splitting (red circle); scale bar: 10 µm. (d) The amplified image of (c); scale bar: 100µm

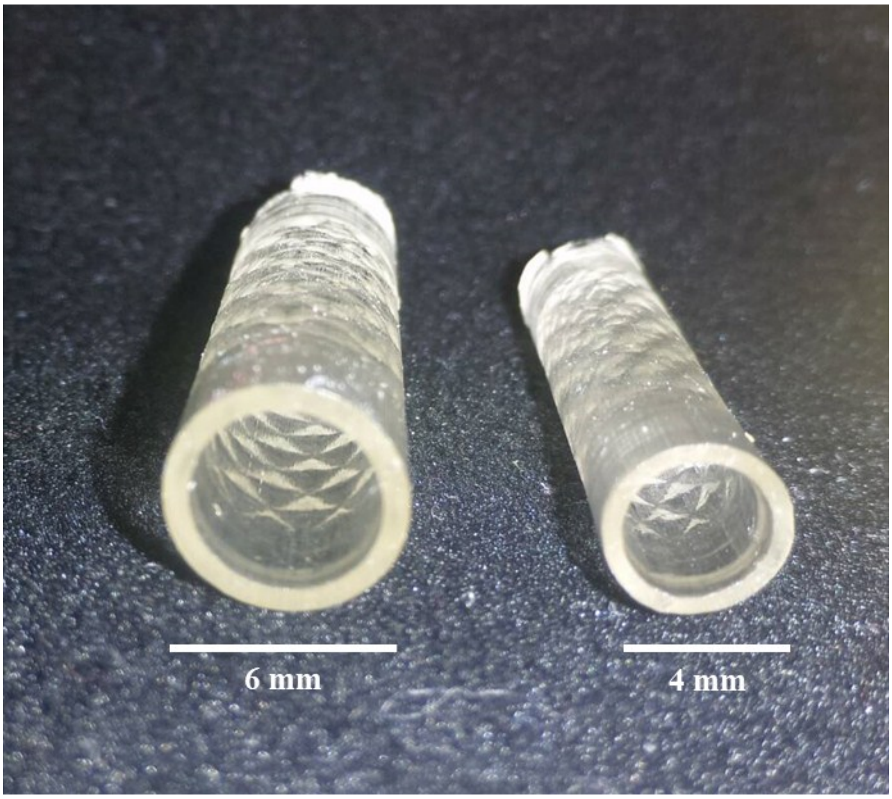

Figure S3. The 3D printed ACMO-AVG with different diameters.

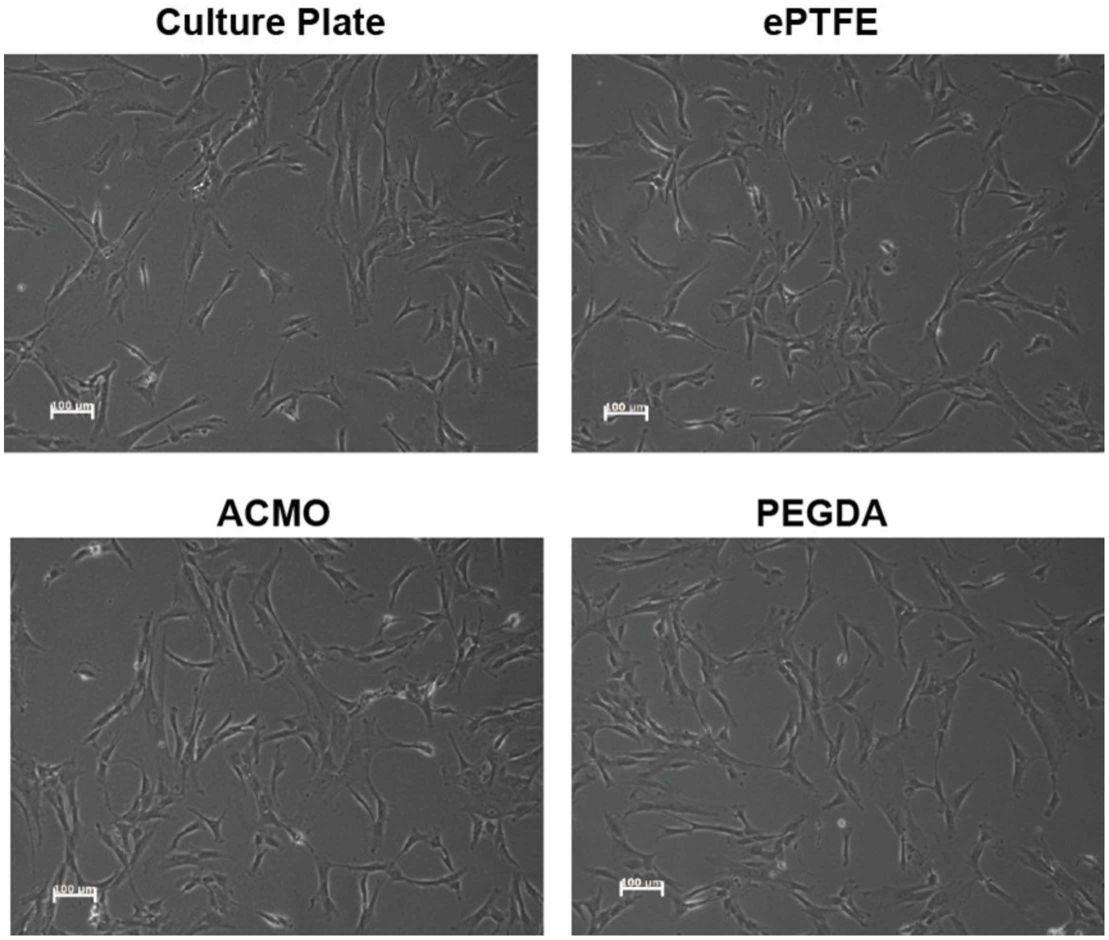

Figure S4. After being incubated in the culture media that had been pre-exposed to different AVG materials for 72 hour, the cell morphology of human skin fibroblast, WS1, in each incubated media was analysed with a light microscope.

Table S1. Summary of the ACMO-AVG with different outer rim design

Outer Rim Design	Splitting occurred during 3D printing	Layer splitting visible under TEM	Structurally stable after 3D printed
Twisted	No	No	Yes
Hexagonal	Yes	Yes	No
Cylindrical	Yes	Yes	No

Table S2. Fracture energy analysis of different AVG expressed as mJ/mm^3

	ePTFE	PEGDA	ACMO
**Tensile Strength**	85.34409	21.18079	146.83013
**Suture Retention**	5.36547	0.24382	3.59071
